# Income Inequality and Self-Serving Belief in Burden-Sharing: An Experimental Study

**DOI:** 10.3390/bs15121689

**Published:** 2025-12-05

**Authors:** Lan Zhou, Xianghong Wang

**Affiliations:** 1 Department of Economics, Hangzhou Normal University, Hangzhou 311121, China; 2 International Business College, South China Normal University, Foshan 528225, China; 3 School of Economics, Renmin University of China, Beijing 100872, China

**Keywords:** inequality, belief distortion, cooperation, fairness norm, self-serving bias

## Abstract

Public goods games under asymmetric endowments have been widely discussed in the literature; however, few studies have addressed how inequality influences normative beliefs and the subsequent burden-sharing behaviors. To address this gap, we conducted two online survey experiments in both hypothetical and real-income scenarios, focusing on the mediation effects of self-serving bias and other-regarding preferences. The findings showed that while unequal endowment status induced self-serving personal beliefs and burden-sharing behaviors, it also enhanced reciprocity and offset self-serving bias in a real-income scenario. Only high-endowment status significantly influenced beliefs and behaviors. This study reveals a trade-off between self-serving bias and reciprocity in social cooperation, offering new insights for fairness beliefs.

## 1. Introduction

From the persistent dialog over progressive taxation to the negotiations on international climate agreements, a fundamental dilemma persists: how should the burdens of financing public goods be equitably shared among individuals with different economic means? These collective action failures frequently arise from conflicting perceptions of fairness (McCaffery & Baron, 2004; Goeschl et al., 2020). Previous studies have extensively investigated public goods experiments under asymmetric endowments, demonstrating that initial wealth disparities have a negative impact on contribution behaviors (e.g., Zelmer, 2003; Buckley & Croson, 2006; Cherry et al., 2005). The influence of real-world income inequality yielded contradictory findings: while a significant portion of the literature points to its detrimental effects on collective action (Zhang et al., 2022), other studies concluded that income inequality can foster greater social sympathy (Mastromatteo & Russo, 2017). The previous literature primarily focused on mechanisms such as reference-dependent preference and inequality aversion (Fehr & Schmidt, 1999; Martinangeli & Martinsson, 2020). However, few studies have explored how inequality might influence individuals’ fairness beliefs. To address the gap, we examined whether income inequality induces self-serving personal and social normative beliefs and consequently reduces cooperative decisions.

Individuals in public goods games derive utility from monetary payoffs and psychological components, such as reciprocity and guilt aversion (Dufwenberg et al., 2011). The utility level relies on individuals’ first-order beliefs (beliefs about others’ actions) and second-order beliefs (whether the contribution level falls short of others’ expectations) (Krupka & Weber, 2013). When others’ conduct deviates from one’s personal norm, a reciprocally motivated individual consequently reduces cooperation, while guilt aversion compels individuals to contribute at least up to the level they believe in others’ expectations (Rabin, 1993; Battigalli & Dufwenberg, 2022). Income inequality influences individuals’ preferences and beliefs, such as procedural fairness and social norms (Tucker & Xu, 2023). Recent cross-national survey experiments suggested that different income groups hold beliefs that the high-income group is less moral and act according to this belief (Pittarello et al., 2022; Almas et al., 2022). According to the theory, we propose the following hypothesis:

**Hypothesis 1** **(H1).** *In contexts of unequal endowments, an individual’s role as allocator will result in burden-allocation behaviors that directionally align with their personal norms and social normative beliefs regarding the fairness of responsibility distribution.*

Belief formation can be demonstrated through two pathways: self-serving bias and other-regarding preferences. On the one hand, self-serving bias typically manifests as an unconscious, yet potent, tendency to internalize and articulate fairness views that inherently align with one’s own economic interests (Dorin et al., 2023). In unequal settings, low-endowment individuals’ preference for redistribution remains consistent with their position behind the veil, whereas high-endowment individuals face a conflict between impartiality and self-interest, prompting a strategic reassessment of fairness (Kriss et al., 2011; Gallier et al., 2016). On the other hand, status comparation will heighten awareness of income inequality, activating other-regarding preferences such as reciprocity and inequality aversion (Dufwenberg et al., 2011; Fehr & Schmidt, 1999), which may suppress the expression of self-serving beliefs. The net effect is an open empirical question, leading to two competing hypotheses.

**Hypothesis 2a** **(H2a).** *If self-serving bias dominates, unequal endowments lead to a self-serving distortion of personal norms, especially for the high-endowment individuals.*

**Hypothesis 2b** **(H2b).** *If other-regarding preferences dominate, unequal endowments lead to a prosocial adjustment of personal norms, especially for the high-endowment individuals.*

Individuals project their personal beliefs onto their peers, making consistent adjustments to their beliefs in social norms according to their personal beliefs under inequality (Loewenstein et al., 2003). This is consistent with the theory of motivated reasoning in belief formation, wherein individuals overestimate the prevalence of their own views in the population (Benabou & Tirole, 2006). Therefore, we propose the following hypothesis:

**Hypothesis 3** **(H3).** *Under unequal endowments, social normative beliefs and personal beliefs will exhibit aligned shifts regarding the fairness of burden-allocation behaviors.*

If self-serving bias dominates, unequal endowments lead to a self-serving distortion of social normative beliefs. If other-regarding preferences dominate, high-endowment individuals expect the other players to endorse high agreement with the progressive proportion principle and low agreement with the equal amount principle.

We conducted two online survey experiments to examine the influence of income inequality on fairness beliefs and to identify the underlying mechanisms. The first experiment employed a manipulated endowment scenario in a hypothetical public goods game to testify whether beliefs and burden-allocation behaviors vary merely with endowment levels. Those in the treatment group were randomly assigned either a high-endowment or low-endowment status, while the control group received no endowment specification. The second experiment used the technique of information intervention to examine the effect of individuals’ actual income status. Participants were instructed to read the same public goods scenario, report their personal and social normative beliefs, and act as uninvolved third-party allocators. Both experiments concluded that income inequality causes self-serving personal beliefs and results in more altruistic behaviors. We further calculated the trade-off between self-serving bias and other-regarding preferences in the pathway of belief formation and demonstrated the effectiveness of reciprocity in counteracting self-serving bias in real-income treatments.

## 2. Experiment 1

### 2.1. Method

#### 2.1.1. Participants

We recruited 614 participants from the Credamo online subject pool. Our target sample size was determined by a power analysis. Setting an effect size of d = 0.25 (a commonly accepted small-to-medium effect size in behavioral economics, reflecting a detectable difference in means between groups) and a significance level of α = 0.05, it theoretically required 159 participants per group to achieve a statistical power (1-β) of 0.8 for detecting the main effect of the endowment manipulation on our key dependent variables.

We implemented a two-stage quality control process. First, we excluded 54 participants (8.8%) with completion times under 5 min, as this represented the fastest 10% of the total sample and fell below the stated 5–10 min consent form threshold, indicating insufficient engagement. Second, we excluded 10 participants who failed a direct attention check question embedded in the questionnaire. This question explicitly instructed participants: “This is an attention check, please answer ‘very dissatisfied’”. The final sample comprised 550 participants.

#### 2.1.2. Design

The study employed a between-subjects experimental design. Participants were randomly assigned to one of three groups that manipulated their assumed initial endowment within a hypothetical public goods game scenario. Importantly, participants made decisions individually, without interacting in real-time four-person groups. The four-person public goods game was presented as a scenario based on a previous actual lab experiment, designed to elicit participants’ perceptions^1^. Three experimental groups were as follows.

- High-endowment scenario (n = 182): Participants were explicitly told to imagine they were assigned to a public goods game scenario with an initial endowment of 40 tokens (e.g., Member 1 in the lab experiment).
- Low-endowment scenario (n = 183): Participants were explicitly told to imagine they were assigned to a public goods game scenario with an initial endowment of 20 tokens (e.g., Member 3 in the lab experiment).
- Baseline scenario (control group, n = 185): Participants were informed about the general structure and endowment heterogeneity (40 vs. 20 tokens) of the public goods game but were not assigned to any specific role or endowment level for themselves. This group served as a neutral benchmark to assess general normative beliefs in the absence of a hypothetical high- or low-endowment experience.

All participants acted as allocators for the four-person group, determining contribution amounts for all members. We employed a non-incentivized belief measurement approach with fixed compensation (CNY 5, ≈USD 0.70), as incentivized methods can introduce a complexity that compromises accuracy in multi-dimensional belief elicitation (Charness et al., 2021). We measured participants’ personal views on burden-sharing principles and their beliefs about social norms.

#### 2.1.3. Materials

- Hypothetical Public Goods Game Scenario

**Figure 1:**
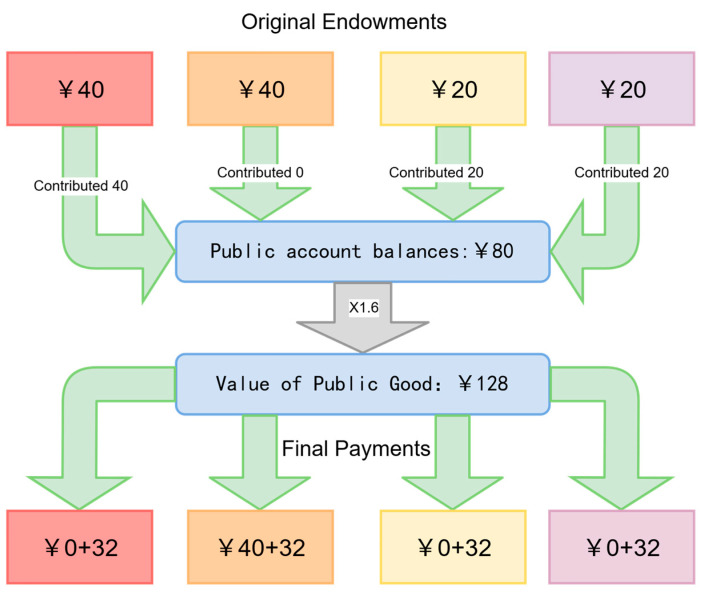
Overview of the public goods contribution scenario used in the survey experiment.

Participants received a detailed description of a one-shot public goods game, framed as a scenario based on an actual laboratory experiment conducted at Renmin University of China. Two members received an initial endowment of 40 tokens and two received 20 tokens. The marginal per capita return (MPCR) was set at 0.4. We provided a concrete example, as illustrated in Figure 1, which explicitly depicted scenarios such as a free-rider outcome where a player starting with 40 tokens contributes nothing yet earns 72 tokens. The parameters were chosen to establish clear and salient inequality, consistent with typical outcomes observed in public goods game experiments. The scenario explicitly stated that participants in the actual lab experiment made anonymous decisions and aimed for payoffs based on the public account, enhancing the realism of the hypothetical context.

- Measures
  1. Allocation behavior: As an involved allocator, participants were asked to hypothetically specify the exact contribution amount (0 to full endowment) for each of the four group members by entering the amount each member should contribute into clearly labeled fields (“Member 1,” “Member 2,” “Member 3,” and “Member 4”). This measure captured participants’ preferred distribution of contributions given the endowment.
  2. Personal beliefs: Participants rated the perceived fairness of three distinct burden-allocation scenarios on a 5-point Likert scale (1 = “very unfair” to 5 = “very fair”): equal amount of contribution, equal proportional contribution, and progressive contribution proportion.
  3. Social normative beliefs: Participants estimated how “most participants in the real experimental scenario” in high-endowment and low-endowment roles would judge the fairness of the same three burden-allocation principles, using the same 5-point scale. They were asked to predict perceptions of high-endowment players and low-endowment players separately^2^.
  4. Control variables: We collected standard demographic data (age, gender, urban/rural residency, education, household income) and subjective views (social trust, self-efficacy), as well as an embedded attention check item. We included measures of other-regarding preferences (reciprocity, perceptions of public goods adequacy, and distributional fairness) adapted from the Global Preferences Survey (Falk et al., 2018).

#### 2.1.4. Procedure

The experiment was administered online via the Credamo platform. Participants were first asked to provide an informed consent and then completed a brief demographic questionnaire. Subsequently, the Credamo system randomly assigned participants to one of three experimental groups: high-endowment, low-endowment, or baseline. Participants in the high-endowment and low-endowment scenarios received explicit information regarding their hypothetical role and initial endowment. They were also clearly informed about the initial endowments of the other three hypothetical group members. In the baseline group, participants received a message indicating an unspecified role, with the focus solely on the general rules of the game.

Following role assignment, all participants read detailed instructions for a hypothetical public goods game. This section comprehensively explained the concept of a public goods game and the game mechanics, including initial endowments, individual contribution decisions, and the marginal per capita return (MPCR), with specific examples. The instructions explicitly contextualized the scenario by stating that approximately 200 university students had participated in the actual game. Next, participants were tasked with deciding the contribution amount to the public account for each of the four hypothetical group members, including the role they were assigned or would have been assigned in the baseline group. This task presented the participant as the sole determinant of contributions for the entire group. After the allocation task, participants proceeded to the belief elicitation phase. They reported their personal beliefs and their social normative beliefs, predicting the fairness judgments of both high-endowment and low-endowment players from the real laboratory experiment.

The experimental session concluded with questionnaires designed to measure control variables. Upon successful completion, participants received a fixed payment.

### 2.2. Results

#### 2.2.1. Descriptive Statistics

Table 1 presents the descriptive statistics for the allocation behaviors and the major control variables. We analyzed behavioral outcomes in two ways: the average contribution amount assigned by participants (in the role of group allocator) to the hypothetical high-endowment and low-endowment players (*hcontri* and *lcontri*); the allocated relative burden *rate* = [100 × (*hcontri*/*lcontri*)](%).

Descriptive results show that endowment status significantly influenced burden-allocation decisions. Compared to the baseline group, participants in the high-endowment scenario allocated significantly lower contribution burdens to high-endowment members (β = −2.897, *p* < 0.01), while their allocations to low-endowment members remained statistically unchanged (β = −0.064, *p* = 0.924). One-way ANOVA tests revealed statistically significant imbalances in age and household income across the treatment groups. To ensure the reliability of the estimated treatment effects, age and household income were included as control variables in all subsequent regressions.

#### 2.2.2. Endowment Status and Normative Beliefs

**Figure 2:**
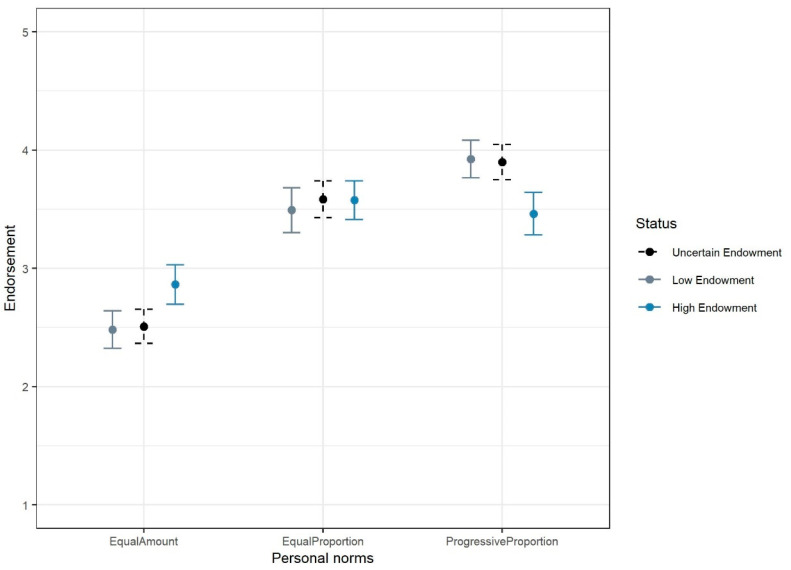
Personal normative beliefs in different treatments. Note: The figure displays mean ratings and 95% confidence intervals for the perceived fairness of three burden-sharing principles: allocating an equal absolute amount, equal proportion, or progressive proportion of contribution to a public good.

Figure 2 presents the means and 95% confidence intervals of participants’ personal normative beliefs under different endowment scenarios, specifically reporting fairness judgments toward three burden-allocation principles. Table S1 in Supplementary Materials S2 reports the regression results of endowment status on personal beliefs^3^. The results indicate that, compared to the baseline condition, participants assigned to the high-endowment scenario showed significantly stronger moral approval for the equal amount principle ($\beta_{high\_endow}=$ 0.377, *p* < 0.01) and lower approval for the progressive proportion principle ($\beta_{high\_endow}=$ −0.390, *p* < 0.01). In contrast, assignment to the low-endowment scenario did not significantly alter normative judgments, since the fairness perceptions were statistically indistinguishable from those in the random-endowment scenario ($\beta_{low\_endow}=$ −0.066, *p* = 0.204 for equal amount norm; $\beta_{low\_endow}=$ −0.035, *p* = 0.465 for equal proportion norm; $\beta_{low\_endow}=$ 0.017, *p* = 0.597 for progressive proportion norm). These findings align with Hypothesis 2a.

**Figure 3:**
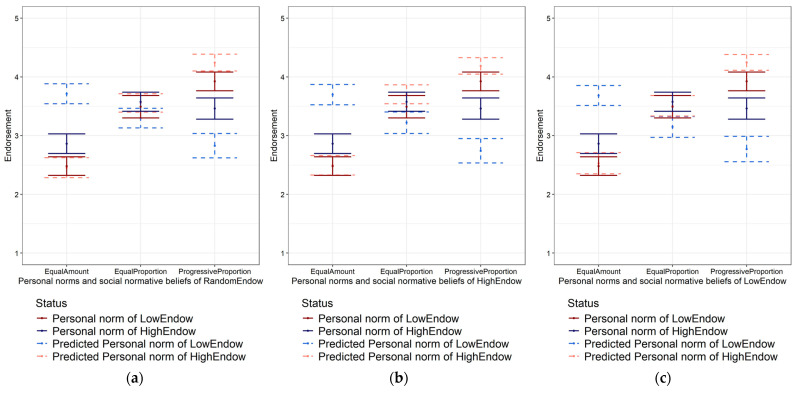
Personal norms and predictions for social norms in different treatment groups. (**a**) Personal norms and social normative beliefs in baseline group; (**b**) personal norms and social normative beliefs in treatment of high-endowment scenario; (**c**) personal norms and social normative beliefs in treatment of low-endowment scenario.

Figure 3 displays the social norms of individuals in both high- and low-endowment scenarios, as well as their predictions of social normative beliefs for each endowment group. All participants expected low-endowment individuals to perceive the equal amount principle as fairer (rank sum test *p* < 0.01) and high-endowment individuals to regard the progressive proportion principle as fairer (rank sum test *p* < 0.01), while the income status showed no significant impact on social beliefs (the *p*-values for between-group differences for social normative beliefs are $p_{EqualAmount\_High}$ = 0.985, $p_{EqualProportion\_High}$ = 0.517, $p_{ProgressiveProportion\_High}$ = 0.805, $p_{EqualAmount\_Low}$ = 0.488, $p_{EqualProportion\_Low}$ = 0.368, and $p_{ProgressiveProportion\_Low}$ = 0.713 from the one-way ANOVA test). Contrary to Hypothesis 3, participants accurately predicted differences in social norms across endowment scenarios, rather than projecting their personal norms.

#### 2.2.3. Mediating Effects: Trade-Off Between Self-Serving Bias and Other-Regarding Preferences

**Figure 4:**
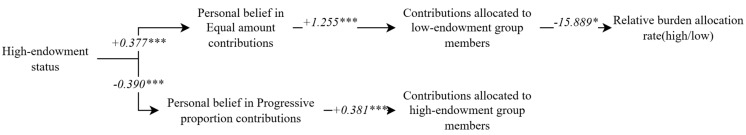
Mediating pathways of hypothetical endowment status on allocation behavior via normative beliefs. Note: Each arrow illustrates a regression relationship. The insignificant pathways are eliminated in the figure. The variable at the arrow’s origin is the explanatory variable, and the variable at its end is the dependent variable. The number on each arrow denotes the regression coefficient. In all regressions shown, if personal beliefs in “Equal amount contributions”, “Equal proportion contributions (eliminated in the figure due to insignificance)” and “Progressive proportion contributions” are the explanatory variables (arrow’s origin), the other variables from this set are included in the same model. * *p* < 0.1, *** *p* < 0.01.

Figure 4 visually summarizes the mediating pathways through which endowment status influences allocation behavior via normative beliefs. We calculated variance inflation factors (VIF) for the respective regression models, obtaining a VIF of 1.86 for the model including only personal belief measures and a VIF of 1.85 for the model incorporating both personal and social normative belief measures. Following the conventional threshold that VIF values below 5 indicate no severe multicollinearity, key explanatory variables do not suffer from problematic multicollinearity in the regression (O’brien, 2007).

First, we testified the influences of endowment status on burden-allocation behaviors. Compared to the baseline group, individuals in the low-endowment scenario assigned significantly higher contribution burdens to both high- and low-endowment members ($\beta_{low\_endow}=$ 1.666, *p* < 0.1 for *hcontri*; $\beta_{low\_endow}=$ 1.201, *p* < 0.01 for *lcontri*). In contrast, individuals in the high-endowment scenario significantly reduced contributions assigned to high-endowment members while leaving contributions to low-endowment members unchanged ($\beta_{high\_endow}=$ −3.224, *p* < 0.01 for *hcontri*; $\beta_{high\_endow}=$ −0.029, *p* = 0.888 for *lcontri*). In terms of the contribution ratio, individuals in both endowment scenarios reduced the relative public goods burden assigned to high-endowment players ($\beta_{low\_endow}=$ −46.743, *p* < 0.01; $\beta_{high\_endow}=$ −43.504, *p* < 0.01 for *lcontri*). Given that Figure 4 focuses on mediating effects and due to the non-significant impact of low-endowment status on fairness beliefs, we excluded its direct influence on individual beliefs from the analysis presented.

To investigate the mediating effect, we added personal and social normative beliefs as control variables and conducted regressions on burden-allocation behaviors. The results show that fairness judgments regarding equal amount contributions significantly predicted allocation behaviors. Stronger endorsement of the equal amount norm was associated with higher contributions assigned to low-endowment members ($\gamma_{norm\_EqualAmount}=$ 1.255, *p* < 0.01 for *lcontri*) and a significantly lower contribution ratio between high- and low-endowment members ($\gamma_{norm\_EqualAmount}=$ −15.889, *p* < 0.1 for *rate*). After controlling for personal beliefs, the coefficient of high-endowment status on the contribution ratio decreased from $\beta_{high\_endow}=$ −43.504 to $\beta_{high\_endow}^{\prime }=$ −31.821 (*p* < 0.01 for both regressions), indicating that the endowment manipulation influenced allocation behavior partly by altering personal norms.

Finally, we examined the potential trade-off between self-serving bias and other-regarding preferences. We conducted regressions of endowment status on other-regarding preferences and then added proxy variables of other-regarding preferences as extra controls. The results are listed in Table S2. The results indicate that other-regarding preferences do not significantly mediate the effect of endowment status on personal beliefs, specifically for reciprocity (β=−0.088, *p* = 0.296), views on public goods adequacy (β=−0.079, *p* = 0.346), and distributional fairness (β=−0.059, *p* = 0.299), in real-world scenarios. Hypothesis 2b was denied. The findings in this section indicate that hypothetical income status in the lab intervention primarily altered burden-allocation behavior by influencing personal beliefs. Among the psychological mechanisms affecting these belief changes, self-serving bias outweighed other-regarding preferences.

## 3. Experiment 2

### 3.1. Method

#### 3.1.1. Participants

We recruited 204 participants from the Credamo online subject pool, with the target sample size determined by the same power analysis to be 101. We implemented the same two-stage quality control process, excluding 20 participants (9.8%) with the fastest completion time, as well as those who failed the attention check. The final sample comprised 184 participants.

#### 3.1.2. Design

For Experiment 2, we conducted an online experiment on 10 October 2023. The online questionnaire was administered to a randomly selected sample from Credamo’s subject pool. All participants received a fixed payment of CNY 5 upon completion of the survey and after passing manual quality checks. Building on the basic procedure of Experiment 1, Experiment 2 omitted the procedure of hypothetical group identity description and incorporated an information intervention using individuals’ income status in real life.

Participants in the treatment group was asked to report their personal annual income for 2022 and were first prompted to estimate the percentile of their personal income within the national population^4^. They were then shown a ladder graph indicating their actual relative income position and whether their initial estimate was accurate^5^. To verify the effectiveness of the intervention, participants were again asked about their perceived income rank and their certainty after the information was provided. Individuals with inaccurate self-perceptions of income status were excluded from the final sample.

In the control group, questions about income level and relative status perception were placed at the end of the questionnaire, after the burden-allocation task and belief elicitation tasks. The remaining procedure aligned with that of Experiment 1.

#### 3.1.3. Measures

The key independent variables in Experiment 2 are whether individuals were treated with the intervention with information about the actual income status (*info*). The information of status is a quintile measure ranging from 1 to 5. An interaction term between the information intervention and status is also included in the regression analyses (*info* × *status*).

We also focus on the subjective variables of personal norm and social normative belief in Experiment 2, as well as the behavioral variable of individuals’ allocation decision in the burden-allocation task. Control variables included demographic characteristics, household economic indicators, and attention measures. Mechanism variables including reciprocity and views on the adequacy and distributional fairness of social public goods were all defined and measured consistently with Experiment 1.

Table 2 presents the sample sizes for each treatment group in Experiment 2, along with descriptive statistics for the main demographic variables. A one-way test indicated that the groups were largely balanced across the observed characteristics, though some age differences were detected. These variables were controlled for in subsequent data analyses.

### 3.2. Results

#### 3.2.1. Information on Real Income Status and Normative Beliefs

**Figure 5:**
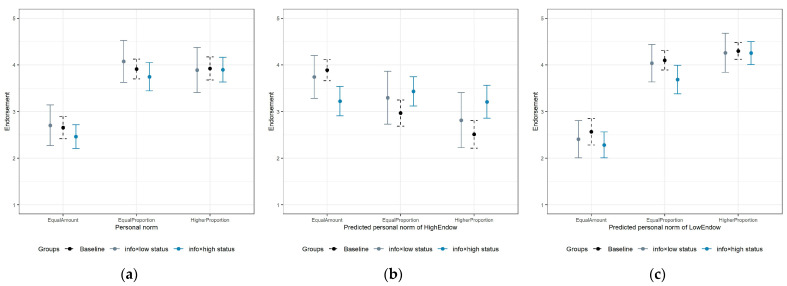
Personal norms and social normative beliefs in experimental treatments. (**a**) Personal norms of individuals in the baseline group and treatment group; (**b**) beliefs in social norms of high-endowment players, obtained from individuals in the baseline group and treatment group; (**c**) beliefs in social norms of low-endowment players, obtained from individuals in the baseline group and treatment group.

Figure 5 displays the average levels of personal and social normative beliefs in both the control and treatment groups. Within the treatment group, we classify participants with middle income and below as the low-SES group (*info* × *low status*) and those with upper-middle and high income as the high-SES group (*info* × *high status*). The results across the three groups indicate that the information intervention with actual income levels has no significant influence on either personal norms or social normative beliefs.

In Table 3, we incorporate control variables and perform ordered-probit regressions. The results show that after priming higher income levels, individuals exhibited increased altruism without significant changes in their personal norms, while their anticipated social norms showed significantly stronger support for the progressive contribution principle ($\beta_{info\times status}$ = 0.395, *p* < 0.05 for predicted personal norm of HighEndow with progressive proportion contribution).

#### 3.2.2. Mediating Effects with Information on Real Income Status

We conducted the mediation analysis based on the pathways in Figure 4. We first analyzed the regression effect of the information intervention on behaviors. The results show that the information intervention for high- or low-income status has no significant influence on burden-allocation behaviors ($\beta_{rate\_allocate}=$ 25.052, *p* = 0.335 for *info × high-income status*; $\beta_{rate\_allocate}=\;-$67.610, *p* = 0.479 for *info × low-income status*).

Second, we investigated the process of belief formation, specifically discussing whether the trade-off between other-regarding preferences and self-serving bias influences personal and normative beliefs. The regression results are listed in Table S3 in the Supplementary Materials. The results indicate that the mediating pathway through reciprocity operated in the opposite direction to the direct treatment effect. Providing information in the high-income group significantly increased individuals’ reciprocity level ($\beta_{reciprocity}$ = −0.268, *p* = 0.070). This, in turn, decreased their personal endorsement for the “Equal Amount” principle (β = −0.238, *p* = 0.010) while increasing support for the “Equal Proportion” principle (β = +0.242, *p* < 0.01). Conversely, after controlling for the proxy variables for other-regarding preferences, the direct treatment effect increased high-income individuals’ endorsement for the “Equal Amount” principle (β = −0.139, *p* = 0.058) while decreasing support for the “Equal Proportion” principle (β = +0.443, *p* = 0.010). Mechanism analysis confirms that both the self-serving bias and other-regarding preferences posited in Hypotheses 2a and 2b are operative in belief formation. While the direct treatment effect exhibited a self-serving bias, reciprocity exerted a cooperative countervailing influence, which together resulted in the non-significant net treatment effect reported in Section 3.2.1.

We further testified whether the information intervention changed personal beliefs in a prosocial way by offering unintended information for the income distribution. The results show that providing information on the income distribution and emphasizing individuals’ high-income status increased their belief in the fairness of the real-world public goods distribution ($\beta_{distribution}$ = −0.580, *p* < 0.01), which in turn increases the personal endorsement for the “Equal Amount” principle (β = +0.263, *p* = 0.029) while decreasing support for the “Equal Proportion” principle (β = −0.361, *p* < 0.01). The information intervention collectively reinforced a self-serving orientation in personal normative beliefs.

## 4. Discussion and Conclusions

This study investigated the influence of income inequality on individuals’ normative beliefs and burden-sharing decisions in public goods cooperation, discussing the trade-off between self-serving bias and other-regarding preferences in belief formation. In Experiment 1, we assigned participants a hypothetical income status within a four-member public goods game scenario featuring unequal endowments (40, 40, 20, 20). Participants assigned to the high-endowment status significantly shifted their personal beliefs regarding the public goods’ burden-allocation principle. They showed a higher endorsement of “contributing equal amounts” and less agreement with “contributing progressive proportions,” thereby lowering the relative burden allocated to high-income group members when acting as group allocators. In the mediation analysis, we observed that unequal hypothetical endowments did not effectively foster other-regarding preferences during belief formation. Consequently, self-serving bias dominated, manifesting in both personal beliefs and behaviors. Hypothesis 1 and Hypothesis 2a were supported, while Hypothesis 3 was rejected. In Experiment 2, we manipulated participants’ awareness of their income status through an information intervention aligned with their actual income. Contrary to Experiment 1, providing information about income status did not significantly influence individuals’ personal or social beliefs, nor did it significantly affect their burden-allocation behaviors. Mediation analysis revealed that belief formation involved two countervailing mechanisms: other-regarding preferences and a direct treatment effect. Specifically, high-income status induced greater reciprocity, increasing endorsement for “contributing equal proportions” while decreasing endorsement for “contributing equal amounts.” However, the parallel direct treatment effect from high-income status exhibited self-serving bias, thereby negating any net influence on beliefs. The combined results of Experiment 1 and Experiment 2 supported a trade-off between H2a and H2b.

### 4.1. General Discussion

The main findings in this study reveal a distinct asymmetry: individuals assigned to high-endowment roles tend to adjust their personal beliefs to favor fairness principles that minimized their risk. In contrast, the personal norms of those in low-endowment roles remained stable. This pattern resonated with the philosophical insight from Rawls’s theory of justice. Our findings suggest that assigning participants to a high-income status essentially lifted the veil of ignorance concerning socioeconomic position. Under such conditions, individuals no longer prioritized the welfare of the least advantaged, but instead exhibited a self-interested preference for maximizing their own benefits (Rawls, 1971). Consistent with the documented divergence in public goods burden-allocation principles, our study provided evidence that high-income individuals implicitly endorse “contributing progressive proportions.” This orientation may be ascribed to the multifaceted nature of fairness norms, subsequently imposing further limitations on the expected contributions of low-endowment individuals (Momeni, 2021).

The mediation analyses further clarified that personal norm served as the primary channel through which endowment status influenced allocation behavior. This result is consistent with Amasino et al. (2023), who found personal norms were more affected by status than social norms in dictator games. This finding also suggests that while social norms may reinforce personal normative adjustments, their direct influence on behavior was limited. This attenuated role could stem from greater perceptual uncertainty in heterogeneous endowment environments regarding the strength and prevalence of social norms. Nevertheless, we acknowledge that higher-order beliefs can be powerful in real-world scenarios (Rockenbach et al., 2021). Our findings highlight the need for theoretical models to carefully delineate the interplay between personal and social norms under inequality.

Finally, mediation analysis in both experiments revealed a trade-off between self-serving bias and other-regarding preferences in the pathway of belief formation. Direct treatment effects stemming from hypothetical or real-world high-income status consistently showed a self-serving tendency. However, information regarding real-world income status simultaneously triggered anticipated reciprocity. These findings offer potential explanations for the inconsistent conclusions in the literature regarding how income inequality influences redistribution preferences. The previous literature also concluded that information on real-income and income distribution may trigger broader social sympathy and explains that self-serving bias may originate from greater psychological distance among different social groups (Mastromatteo & Russo, 2017).

This study offers some policy implications. First, given the pronounced self-serving bias in personal norms among the advantaged, policies could benefit from establishing clear, explicit contribution guidelines. Second, fostering a social consensus around equitable contributions, particularly from advantaged groups, could leverage social influence to encourage more cooperative behavior.

### 4.2. Limitations and Future Research

Several limitations of this study can be acknowledged. First, the cultural context (China) and the use of an online platform (Credamo) may affect the generalizability of our findings. Cultural norms regarding inequality and platform-specific participant pools could yield different results in other settings. Future cross-cultural replications and laboratory studies are warranted. Second, our sample, particularly in Experiment 1, was highly educated, which may limit the representativeness of the results. Future research should strive for more diverse samples to assess the robustness of our findings across different demographic groups. Third, while we used a non-incentivized method for belief elicitation for compelling methodological reasons and such methods often yield comparable results to incentivized ones (Charness et al., 2021), this choice might still be seen as a limitation regarding behavioral external validity. Finally, our study captured short-term responses. Longitudinal research tracking the evolution of normative beliefs through repeated interactions and investigations into how early-life socioeconomic status shapes long-term cooperative dispositions would be valuable extensions.

## Figures and Tables

**Table 1:** Descriptive statistics of low-endowment, high-endowment, and baseline treatment groups in Experiment 1.

Treatment	Low-Endowment Status		High-Endowment Status		Random Endowment (Baseline)		*p*-Values
	N = 183		N = 182		N = 185		
Variables *	Mean	sd	Mean	sd	Mean	sd	
*hcontri*	22.385	9.707	17.708	9.072	20.605	9.669	0.597
*lcontri*	10.333	6.321	8.971	6.098	9.035	6.605	0.558
*rate*	257.609	148.302	267.017	168.751	307.799	179.427	0.040
*age*	2.628	1.155	2.533	1.08	2.443	0.859	0.000
*ifmale*	0.448	0.499	0.407	0.493	0.384	0.488	0.955
*urban*	0.503	0.501	0.637	0.482	0.486	0.501	0.833
*edu*	0.842	0.366	0.819	0.386	0.881	0.325	0.059
*ln_income*	10.76	1.272	10.521	1.704	10.771	1.39	0.000

Note: *p*-values represent the results of the one-way ANOVA (for continuous variables) comparing means/proportions across the three groups. * Age was categorized into six ranges: 1 (0–20 years), 2 (21–30 years), 3 (31–40 years), 4 (41–50 years), 5 (51–60 years), and 6 (over 60 years). Educational attainment (edu) was dichotomized: 0 for primary school or below through junior college, and 1 for a bachelor’s degree or higher.

**Table 2:** Descriptive statistics of sample in baseline and information intervention groups in Experiment 2.

Treatment	Baseline (Info = 0)		Information Intervention (Info = 1)		*p*-Values
	N = 90		N = 94		
Variables	Mean	sd	Mean	sd	
*age*	2.889	0.929	3.096	1.219	0.011
*ifmale*	0.478	0.502	0.426	0.497	0.921
*status*	3.8	1.552	3.766	1.562	0.953
*urban*	0.711	0.456	0.67	0.473	0.730
*edu*	0.833	0.375	0.819	0.387	0.761
*ln_income*	10.858	1.154	10.813	1.288	0.299

**Table 3:** Regressions of information intervention and income status on normative beliefs.

	(1)	(2)	(3)	(4)	(5)	(6)	(7)	(8)	(9)
	Personal Norm			Predicted Personal Norm of HighEndow			Predicted Personal Norm of LowEndow		
Variables	Equal Amount	Equal Proportion	Progressive Proportion	Equal Amount	Equal Proportion	Progressive Proportion	Equal Amount	Equal Proportion	Progressive Proportion
*info × status*	−0.073	−0.193	−0.015	−0.152	0.055	0.395 **	0.175	−0.285 **	−0.092
	(0.081)	(0.135)	(0.059)	(0.108)	(0.080)	(0.122)	(0.113)	(0.063)	(0.046)
*info*	0.202	0.404	0.121	0.270	−0.012	−0.967	−0.971	0.582	0.245
	(0.300)	(0.691)	(0.162)	(0.460)	(0.362)	(0.434)	(0.538)	(0.287)	(0.106)
*status*	−0.050	−0.236	−0.160	−0.336	−0.115	−0.146	−0.406	−0.105	0.066
	(0.170)	(0.255)	(0.215)	(0.176)	(0.059)	(0.123)	(0.251)	(0.202)	(0.133)
Controls	Yes	Yes	Yes	Yes	Yes	Yes	Yes	Yes	Yes
Province Fixed Effect	Yes	Yes	Yes	Yes	Yes	Yes	Yes	Yes	Yes
Observations	184	184	184	184	184	184	184	184	184
R-squared	0.263	0.222	0.263	0.235	0.305	0.244	0.367	0.259	0.258

Robust standard errors in parentheses. ** *p* < 0.05.

## Data Availability

The raw data supporting the conclusions of this article will be made available by the authors on request.

## Notes

1 The situational descriptions presented in our questionnaire were derived from an actual laboratory experiment carried out at Renmin University of China from 2021 to 2022. The questionnaire briefly outlined the experiment’s setup, procedures, and participant numbers, with all sensitive data being excluded.

2 The prompt for this task clarified that, “In the actual experimental game, we also asked all participants the same fairness judgment questions as above. Please estimate, based on the prompt, how these participants would judge the fairness of the above statements.” Participants also reported their certainty about these predictions on a 0–10 scale (0 = “very uncertain” to 10 = “very certain”).

3 The empirical specifications in this study all controlled for demographic characteristics (age, gender, household income, urban residency, education, and relative income), subjective measures (familiarity with common public goods, social trust and self-efficacy), and province fixed effects.

4 Income group classifications were based on the “Statistical Communiqué of the People’s Republic of China on the 2022 National Economic and Social Development” issued by the National Bureau of Statistics of China. Participants were divided into five groups according to their personal disposable income: low income, lower-middle income, middle income, upper-middle income, and high income.

5 Participants were presented with a message such as: “According to the statistical results released by the National Bureau of Statistics, the distribution of per capita disposable income across different income groups in 2022 is shown below. Based on the disposable income level you reported for 2022, your actual position is closest to the low-income group. You accurately estimated your relative income position during the earlier estimation task.” The individual’s actual income group was highlighted in a ladder diagram.

## Supplementary Materials

The following supporting information can be downloaded at https://www.mdpi.com/article/10.3390/bs15121689/s1, Supplementary S1: Theoretical model; Supplementary S2: Supplementary Analyses. Table S1: Oprobit regression of endowment status on personal normative beliefs. Table S2: Mediation analysis for other-regarding preferences in Experiment 1. Table S3: Mediation analysis for other-regarding preferences in Experiment 2.

## References

[B1-behavsci-15-01689] Almas I., Cappelen A. W., Sorensen E. O., Tungodden B. (2022). Global evidence on the selfish rich inequality hypothesis. Proceedings of the National Academy of Sciences of the United States of America.

[B2-behavsci-15-01689] Amasino D. R., Pace D. D., van der Weele J. (2023). Self-serving bias in redistribution choices: Accounting for beliefs and norms. Journal of Economic Psychology.

[B3-behavsci-15-01689] Battigalli P., Dufwenberg M. (2022). Belief-dependent motivations and psychological game theory. Journal of Economic Literature.

[B4-behavsci-15-01689] Benabou R., Tirole J. (2006). Belief in a just world and redistributive politics. Quarterly Journal of Economics.

[B5-behavsci-15-01689] Buckley E., Croson R. (2006). Income and wealth heterogeneity in the voluntary provision of linear public goods. Journal of Public Economics.

[B6-behavsci-15-01689] Charness G., Gneezy U., Rasocha V. (2021). Experimental methods: Eliciting beliefs. Journal of Economic Behavior & Organization.

[B7-behavsci-15-01689] Cherry T. L., Kroll S., Shogren J. F. (2005). The impact of endowment heterogeneity and origin on public good contributions: Evidence from the lab. Journal of Economic Behavior & Organization.

[B8-behavsci-15-01689] Dorin C., Hainguerlot M., Huber-Yahi H., Vergnaud J.-C., de Gardelle V. (2023). How economic success shapes redistribution: The role of self-serving beliefs, in-group bias and justice principles. Judgment and Decision Making.

[B9-behavsci-15-01689] Dufwenberg M., Gächter S., Hennig-Schmidt H. (2011). The framing of games and the psychology of play. Games and Economic Behavior.

[B10-behavsci-15-01689] Falk A., Becker A., Dohmen T., Enke B., Huffman D., Sunde U. (2018). Global evidence on economic preferences. Quarterly Journal of Economics.

[B11-behavsci-15-01689] Fehr E., Schmidt K. M. (1999). A theory of fairness, competition, and cooperation. The Quarterly Journal of Economics.

[B12-behavsci-15-01689] Gallier C., Kesternich M., Sturm B. (2016). Voting for burden sharing rules in public goods games. Environmental and Resource Economics.

[B13-behavsci-15-01689] Goeschl T., Kettner S. E., Lohse J., Schwieren C. (2020). How much can we learn about voluntary climate action from behavior in public goods games?. Ecological Economics.

[B14-behavsci-15-01689] Kriss P. H., Loewenstein G., Wang X., Weber R. A. (2011). Behind the veil of ignorance: Self-serving bias in climate change negotiations. Judgment and Decision Making.

[B15-behavsci-15-01689] Krupka E. L., Weber R. A. (2013). Identifying social norms using coordination games: Why does dictator game sharing vary?. Journal of the European Economic Association.

[B16-behavsci-15-01689] Loewenstein G., O’Donoghue T., Rabin M. (2003). Projection bias in predicting future utility. Quarterly Journal of Economics.

[B17-behavsci-15-01689] Martinangeli A. F. M., Martinsson P. (2020). We, the rich: Inequality, identity and cooperation. Journal of Economic Behavior & Organization.

[B18-behavsci-15-01689] Mastromatteo G., Russo F. F. (2017). Inequality and charity. World Development.

[B19-behavsci-15-01689] McCaffery E. J., Baron J. (2004). Framing and taxation: Evaluation of tax policies involving household composition. Journal of Economic Psychology.

[B20-behavsci-15-01689] Momeni F. (2021). Voluntary and mandatory provision of common-pool resources with heterogeneous users. Journal of Economic Behavior & Organization.

[B21-behavsci-15-01689] O’brien R. M. (2007). A caution regarding rules of thumb for variance inflation factors. Quality & Quantity.

[B22-behavsci-15-01689] Pittarello A., Motsenok M., Dickert S., Ritov I. (2022). When the poor give more than the rich: The role of resource evaluability on relative giving. Journal of Behavioral Decision Making.

[B23-behavsci-15-01689] Rabin M. (1993). Incorporating fairness into game theory and economics. The American Economic Review.

[B24-behavsci-15-01689] Rawls J. (1971). A theory of justice.

[B25-behavsci-15-01689] Rockenbach B., Tonke S., Weiss A. R. (2021). Self-serving behavior of the rich causes contagion effects among the poor. Journal of Economic Behavior & Organization.

[B26-behavsci-15-01689] Tucker S., Xu Y. (2023). Fairness, (perception of) inequality, and redistribution preferences. Journal of Economic Surveys.

[B27-behavsci-15-01689] Zelmer J. (2003). Linear public goods experiments: A meta-analysis. Experimental Economics.

[B28-behavsci-15-01689] Zhang Y., Zuo C., Zhang B. (2022). Income inequality and collective action: Evidence from rural tax-for-fee reform in China. Journal of Agrotechnical Economics.

