# Supplementary material for: Income Inequality and Self-Serving Belief in Burden-Sharing: An Experimental Study
Source: Behav Sci (Basel). 2025 Dec 5;15(12):1689. doi: 10.3390/bs15121689 (PMC12729624; doi:10.3390/bs15121689)
Supplement: Supplementary file 1 [file behavsci-15-01689-s001.zip › behavsci-3928079-supplementary.pdf]

Supplementary Materials

S1. Theoretical model

We begin by constructing a theoretical model based on standard psychological game theory. An individual's utility in a public goods game comprises three components: utility from monetary payoff, utility from reciprocity based on both players' actions, and utility loss from failing to meet the opponent's expectations (Rabin, 1993; Battigalli and Dufwenberg, 2022). The baseline utility function of individual i is formulated as follows:

$$u_i = v_i[(E_i - C_i) + MPCR * \sum C_j] + R_i * \sum_{j \neq i} \kappa_{ij} \cdot \lambda_{iji} - G_i \cdot \max\{0, \frac{\sum_{j \neq i} f_{iji}}{N-1} - C_i\} \quad (S1)$$

We denote E_i as the initial endowment available to individual i, C_i as the amount contributed to the public account, and $MPCR$ as the marginal per capita return from the public good.

The first component, $v_i(\cdot)$, represents the utility derived from monetary payoff. We assume $v_i(\cdot)$ exhibits diminishing marginal returns, such that $v_i' > 0, v_i'' < 0$.

The second component introduces concepts of personal fairness norms and subjective beliefs to model reciprocity based on both players' actions and normative beliefs. Here, R_i denotes the intensity of i's reciprocal preference, with a higher R_i indicating stronger reciprocity. κ_{ij} represents i's kindness toward j, defined as the difference between i's contribution level and i's second-order belief. λ_{iji} captures i's belief about j's kindness toward i, defined as the difference between j's contribution and i's first-order belief.

We define beliefs as follows. The **first-order belief**, denoted $f_{ij} \in [f_{ij}^{min}, f_{ij}^{max}]$, is i's expectation regarding j's normatively appropriate contribution level, based on i's own subjective norm or fairness perception. The **second-order belief**, denoted $f_{iji} \in [f_{iji}^{min}, f_{iji}^{max}]$, is i's belief about j's first-order belief regarding i's contribution (i.e., what i believes j expects from i). The second-order belief serves as a reference point for i's contribution.

In an environment with transparent contribution information, if j's actual contribution $\widetilde{C_j}$ falls below i's first-order belief, a reciprocally motivated individual i is inclined to perceive j as uncooperative. This may lead i to contribute less than their second-order belief in subsequent rounds, resulting in

cooperation breakdown. We define the kindness functions as follows:

$$\kappa_{ij} = C_i - f_{iji} \tag{S2}$$

$$\lambda_{iji} = \widetilde{C_j} - f_{ij} \tag{S3}$$

The third component of the utility function captures the utility loss due to guilt aversion. Here, G_i denotes i's degree of guilt aversion. A higher G_i indicates a greater utility loss arising from feelings of guilt. While reciprocally motivated individuals desire to meet their opponent's expectations conditional on the opponent also exhibiting reciprocal tendencies, guilt-averse individuals unconditionally wish their contribution level to align with group members' expectations; failure to do so results in a utility loss.

Belief formation is grounded in personal norms and individuals' perceptions of socially prevalent norms. This study focuses on conceptions of fairness regarding the allocation of public goods responsibilities and the formation of first- and second-order beliefs within the benchmark model. Let r_i represent i's preferred principle for allocating public goods responsibilities under initial endowment inequality(i.e., their conception of fairness). To simplify the analysis, we follow existing theoretical literature by assuming two types of fairness views: equal amount and equal proportion of contribution. Individuals who adhere to the **equal absolute contribution** principle prefer all members to contribute the same absolute amount, i.e., $C_i = C_j$, and in this case, we define $r_i = 0$. Those who hold the **equal proportional contribution** principle prefer all members to contribute the same proportion of their initial endowments, i.e., $C_i/E_i = C_j/E_j$ and here we define $r_i = 1$.

In a public goods game with endowment inequality, relative endowment and personal fairness views jointly determine the interval within which beliefs are formed. To simplify, suppose there are two members with initial endowments E_l and E_h, where $E_l < E_h$. First, an individual's first-order belief depends on their own fairness view: $f_{ij} = f_{ij}(E_i, E_j, r_i)$. For a **high-endowment** individual, they expect their opponent to contribute anywhere within the full range of the opponent's endowment. Thus, the first-order belief interval is $[0, E_l]$. For a **low-endowment** individual, under an equal amount burden sharing norm, the expected fair contribution from the opponent remains $[0, E_l]$. Under an equal proportional view, the first-order belief interval becomes $[0, E_h]$.

Second, individual i forms an expectation about j's fairness view, denoted $Exp_i[r_j]$, and this expected social norm determines the second-order belief: $f_{iji} = f_{iji}(E_i, E_j, Exp_i[r_j])$. If i

is a high-endowment player and expects society to endorse the equal absolute contribution view, they believe that j's first-order belief about i's contribution is equivalent in absolute terms to j's own contribution. Hence, the second-order belief interval is $[0, E_l]$. If i expect society to endorse the equal proportional view, they believe j's first-order belief about i should be proportional to j's contribution. Thus, the second-order belief interval is $[0, E_h]$. For a low-endowment individual i, regardless of whether they expect the opponent to hold an equal absolute or equal proportional fairness view, the second-order belief interval remains $[0, E_l]$.

Following Dufwenberg et al. (2011), we simplify by taking the midpoint of the belief interval. Thus, the first-order belief of a high-endowment individual is constant: $f_{hl}(r_i) = E_l/2$. The second-order belief can be expressed as:

$$f_{hlh} = \begin{cases} E_l/2\,, if\ Exp_i[r_j] = 0 \\ E_h/2\,, if\ Exp_i[r_j] = 1 \end{cases} \tag{S4}$$

In the subjective decision-making process of psychological games, the initial contribution level of high-endowment individuals is centered around their second-order belief as a reference point. For those with reciprocal preferences and guilt aversion, individuals who priorly expect the social norm to be equal proportional contributions ($r_i = 1$) exhibit a higher initial contribution level compared to those who expect a norm of equal absolute contributions ($r_i = 0$).

For low-endowment individuals, the second-order belief remains constant at $f_{lhl}(r_i) = E_l/2$, while the first-order belief is given by:

$$f_{lh} = \begin{cases} E_l/2\,, if\ r_i = 0 \\ E_h/2\,, if\ r_i = 1 \end{cases} \tag{S5}$$

Consistent with existing literature, individuals update their beliefs based on observed contribution information from opponents in repeated interactions. In this setting, we assume that $\widetilde{C_J}$ is updated after each round using the average contribution of other members: $\widetilde{C_J}^t = \sum_{j \neq i} C_j^t / N - 1$. The first-order belief serves as a subjective reference point for judging the kindness of opponents. Contributions above this reference are perceived as signals of cooperation and kindness, whereas those below are seen as indications of selfishness or non-cooperation. If an opponent's contribution exceeds the first-order belief, the individual reciprocates with full contribution in round t to enhance utility from reciprocity; otherwise, no contribution is made. In the reference-dependent behavior of high-endowment individuals, the first-order belief remains constant across

different fairness views, denoted as $f_{hl}(r_i = 1) = f_{hl}(r_i = 0)$, reflecting consistent feedback responsiveness to contribution signals regardless of their normative belief. For low-endowment individuals, we define: $f_{lh}(r_i = 1) = E_h/2 > E_l/2 = f_{lh}(r_i = 0)$. When the observed contribution lies in the interval $[E_l/2, E_h/2]$, the interpretation of the opponent's kindness depends on the individual's fairness view: those with personal normative belief of equal amount perceive contributions in this range as cooperative signals, while those with an equal proportional view consider the same range insufficiently cooperative, leading to lower subsequent contributions compared to the former group.

S2. Supplementary Analyses

Table S1. Oprobit regression of Endowment status on personal normative beliefs.

Variables	(1)<br>norm_EqualAmount	(2)<br>norm_EqualProportion	(3)<br>norm_ProgressiveProportion
low_endow	-0.066	-0.035	0.017
	(0.052)	(0.050)	(0.043)
high_endow	0.377***	0.044	-0.390***
	(0.062)	(0.060)	(0.036)
Controls	Y	Y	Y
Province Fixed Effect	Y	Y	Y
Observations	550	550	550

Robust standard errors in parentheses
**** p<0.01, ** p<0.05, * p<0.1*

Table S2. Mediation analysis for other-regarding preferences in Experiment 1

Variables	(1)<br>Reciprocity	(2)<br>PGG Distributional Fairness	(3)<br>PGG adequacy	(4)<br>norm_ Equal Amount	(5)<br>norm_ Equal Proportion	(6)<br>norm_ Progressive Proportion
low_endow	0.249***	0.039	0.008	-0.053	-0.041	0.034
	(0.047)	(0.051)	(0.064)	(0.048)	(0.055)	(0.043)
high_endow	-0.088	0.059	0.079	0.367***	0.041	-0.426***
	(0.075)	(0.051)	(0.076)	(0.055)	(0.066)	(0.044)
Reciprocity				-0.050	0.031	-0.009
				(0.038)	(0.028)	(0.012)
PGG Distributional Fairness				0.134*	0.054	0.006
				(0.068)	(0.060)	(0.049)
PGG adequacy				-0.156**	0.010	-0.033
				(0.078)	(0.078)	(0.060)
Controls	Y	Y	Y	Y	Y	Y

Province Fixed Effect	Y	Y	Y	Y	Y	Y
Observations	550	550	550	550	550	550
R-Squared	0.112	0.470	0.198			

Robust standard errors in parentheses
**** p<0.01, ** p<0.05, * p<0.1*

Table S3. Mediation analysis for other-regarding preferences in Experiment 2

**Variables**	**(1)** **Reciprocity**	**(2)** **PGG Distributional Fairness**	**(3)** **PGG adequacy**	**(4)** **norm_ Equal Amount**	**(5)** **norm_ Equal Proportion**	**(6)** **norm_ Progressive Proportion**
Panel A: High Income Status						
Info	**0.268***	**0.580*****	0.804***	**0.139***	**-0.443****	-0.121
	**(0.097)**	**(0.153)**	(0.251)	**(0.073)**	**(0.172)**	(0.095)
Reciprocity				**-0.239*****	**0.242*****	0.055
				**(0.092)**	**(0.015)**	(0.094)
PGG Distributional Fairness				**0.263****	**-0.361*****	0.036
				**(0.121)**	**(0.089)**	(0.162)
PGG adequacy				0.085	0.128	-0.141
				(0.194)	(0.254)	(0.130)
Controls	Y	Y	Y	Y	Y	Y
Province Fixed Effect	Y	Y	Y	Y	Y	Y
Observations	129	129	129	129	129	129
R-Squared	0.291					
Panel B: Low Income Status						
Info	0.449	-0.464**	-0.341	-1.184**	1.079	1.047
	(0.244)	(0.194)	(0.497)	(0.467)	(0.969)	(0.694)
Reciprocity				0.043	-0.326	-0.227
				(0.224)	(0.276)	(0.190)
PGG Distributional Fairness				1.089**	-0.144	-0.188
				(0.507)	(0.243)	(0.362)
PGG adequacy				1.274***	-1.033**	-0.044
				(0.299)	(0.526)	(0.239)
Controls	Y	Y	Y	Y	Y	Y
Province Fixed Effect	Y	Y	Y	Y	Y	Y
Observations	55	55	55	55	55	55
R-Squared	0.559					

Robust standard errors in parentheses
**** p<0.01, ** p<0.05, * p<0.1*
